# Maternal factors contributing to under-five mortality at birth order 1 to 5 in India: a comprehensive multivariate study

**DOI:** 10.1186/2193-1801-2-284

**Published:** 2013-06-27

**Authors:** Rajvir Singh, Vrijesh Tripathi

**Affiliations:** Cardiology Research Centre, Heart Hospital, Hamad Medical Corporation (HMC), Doha, Qatar; Department of Mathematics & Statistics, The Faculty of Science and Technology, The University of the West Indies, St Augustine Campus, Kragujevac, Trinidad & Tobago, West Indies

**Keywords:** India, Under-five mortality, Demographic and health survey (DHS), Birth order

## Abstract

The objective of the study is to assess maternal factors contributing to under-five mortality at birth order 1 to 5 in India. Data for this study was derived from the children’s record of the 2007 India National Family Health Survey, which is a nationally representative cross-sectional household survey. Data is segregated according to birth order 1 to 5 to assess mother’s occupation, Mother’s education, child’s gender, Mother’s age, place of residence, wealth index, mother’s anaemia level, prenatal care, assistance at delivery , antenatal care, place of delivery and other maternal factors contributing to under-five mortality. Out of total 51555 births, analysis is restricted to 16567 children of first birth order, 14409 of second birth order, 8318 of third birth order, 5021 of fourth birth order and 3034 of fifth birth order covering 92% of the total births taken place 0–59 months prior to survey. Mother’s average age in years for birth orders 1 to 5 are 23.7, 25.8, 27.4, 29 and 31 years, respectively. Most mothers whose children died are Hindu, with no formal education, severely anaemic and working in the agricultural sector. In multivariate logistic models, maternal education, wealth index and breastfeeding are protective factors across all birth orders. In birth order model 1 and 2, mother’s occupation is a significant risk factor. In birth order models 2 to 5, previous birth interval of lesser than 24 months is a risk factor. Child’s gender is a risk factor in birth order 1 and 5. Information regarding complications in pregnancy and prenatal care act as protective factors in birth order 1, place of delivery and immunization in birth order 2, and child size at birth in birth order 4. Prediction models demonstrate high discrimination that indicates that our models fit the data. The study has policy implications such as enhancing the Information, Education and Communication network for mothers, especially at higher birth orders, in order to reduce under-five mortality. The study emphasises the need of developing interventions to address the issues of anaemia, mothers working in the agricultural sector and improving relevant literacy among mothers.

## Introduction

Globally, the under-five mortality rates have declined from 85 per 1000 to 51 per 1000 (UNICEF 2012). However, it is estimated that more than 7 million children will die before attaining the age of five. Of these, India, Pakistan, Ethiopia, Nigeria and Democratic Republic of Congo will suffer half of all under-five children deaths (UNICEF 2008). India alone shares the burden of 24% of world’s under-five mortality followed by Nigeria which shares 11% of this burden (UNICEF 2012). It is obvious that health policies in these five countries need to be reviewed and new impetus provided to bring down the high under-five mortality rate. India has seen a decline in these rates from 124 per 1000 in 1990 to 61 per 1000 in 2011 (UNICEF 2012). However, as part of its commitment to reduce under-five mortality to 41 per 1000 by 2015 (UNICEF 2012), India needs to become a special focus area.

The United Nations Children’s Fund (UNICEF) identifies that children are at a greater risk of dying before age five if they are born in rural areas, among the poor, or to a mother deprived of basic education (UNICEF 2012). Even though the National Family Health Survey in India (NFHS-3 2007) highlights that the rate of reduction in under-five mortality rates has been higher in rural areas compared to urban areas, these figures are qualified by wealth, educational and gender inequalities (NFHS-3 2007). Moreover, the fact that under-five mortality is not declining at a faster rate points to policy focus areas not yet under coverage. A major cause of concern is the stagnation in reduction of neonatal deaths (Bhaumik 2013; Kumar et al. 2013). Though it has been pointed out that there is need for “investment in new-born care units and intensive care units” (Bhaumik 2013), there is also need to strengthen maternal services for the lengthy prenatal period. This study is conceived to find the socio-demographic factors related to maternal health care. The study aims to identify if the determinants of under-five mortality change according to birth order.^a^ While birth order plays the role of a strong confounder in under-five mortality, Srinivasan postulated that the intrinsic growth rate and the mean generational length of any population may get affected by the birth order pattern (Srinivasan 1980). For this reason, stratification according to birth order is necessary to properly understand patterns of epidemiological predictors of under-five mortality. Detailed analysis can lead to identification of concerns specific to each birth order since primagravidae are the most at risk. Using birth order as the basis of segregation can have policy implications and reveal specific populations to be targeted for extension of information, education and communication (IEC). Moreover, the World Health Organisation recognises reproductive health as inclusive of “the right of access to appropriate health-care services that will enable women to go safely through pregnancy and childbirth and provide couples with the best chance of having a healthy infant (Glasier et al. 2006)”. Hence, it is important to recognise target populations to customise maternal health care in spite of inequalities of rural urban, wealth, gender and education to ensure the health of mother and child with the aim of reducing under-five mortality.

## Materials and methods

### Data

Data file IAKR52FL.SAV on children records was accessed from Demographic and Health Survey (DHS) site with due permission. The sample was weighted for population differences, rural–urban and slum and non-slum proportional differences to arrive at a nationally representative data in NFHS- 3 conducted on the DHS format used across 80 countries (NFHS-3 2007). The nationally representative household based sample was created through stratified, multistage clustering sampling strategy with two stages at rural level and three stages at urban level. Information on children based on mother’s self-report of birth and death history of children born are included in the study from records of all interviewed women 15–49 years in 29 states in 6 regions (NFHS −3 2007). A total of 51555 of first to fifteenth birth order occurred in the period. The average birth order of children in the sample was 2.6 ± 1.8. Hence, it was decided to restrict the study from birth order 1 to 5. A final total of 16567 children of first birth order, 14409 of second birth order, 8318 of third birth order, 5021 of fourth birth order and 3034 of fifth birth order capturing 92% of the total births during 59 months preceding the survey are available for the analysis. In case of multiple births, only first birth was included in the analysis (NFHS −3 2007).

### Explanatory variables

Mother’s age at index child in years, gender of child at index birth order (male, female), place of residence (rural, urban), religion (Hindu, Muslim, Christian and others), type of caste (scheduled caste, scheduled tribe, other backward classes and Others), standard of living (low, medium, and high), mother’s occupation (non-working, service and agriculture), mother’s anaemia level (no anaemia, severe, moderate, and mild), prenatal care (yes, no), assistance at delivery (yes, no), antenatal care (home, govt. hospital, private hospital and village/NGO), information regarding complications in pregnancy (yes, no), pregnancy health nutrition education (yes, no), Breastfeeding health nutrition education (yes, no), place of delivery (home, hospital /others), breastfeeding (yes, no), immunization (yes, no), child’s age in months (<1 month, 1–12 months and > 12 months), birth interval (< 24 months, 24–36 months and > 36 months) and region ( North, Central, East, North East, West and South) are all taken as explanatory variables as defined by the NFHS-3. For purposes of univariate and multivariate analysis, a few variables are modified/ merged into lesser categories for better understanding of results. Wealth index (poorest, poorer, middle, richer and richest) is recoded as poorest and poor, middle and richer and richest. Mother’s and father’s education is coded as no formal education and primary, secondary and greater than higher secondary. Father’s occupation (non-working, service and agriculture) is taken as part of the definition of the socio-economic status of the family. Size of child at birth (very large, larger than average, smaller than average and very small) are recoded as larger than average, average and smaller than average. All these variables are taken as independent variables and child’s status (live or dead) as dependent variable in the study. Child’s age is calculated as difference in months between the date of birth and the date of interview for living child and between the date of birth and the date of death of child as reported by the mother (NFHS-3 2007).

### Statistical analysis

Frequency tabulations describe the characteristics of under-five children at each birth order in the study. It is observed that most of the deaths in the data occur within one month or at the time of birth. Cox Proportional Hazard assumptions assume a linear relationship exists between the endpoint and predictor variables. Further, recall bias would be present in the data due to the retrospective nature of the data collection. Due to non-fulfillment of assumptions and the possibility of recall bias in the current data set that could cause misleading estimates, logistic regression is chosen as the appropriate statistical tool for analysis. Logistic regression is used to determine factors associated with the mortality forcing child age in months and mother’s age at index child into multivariate analysis at each birth order as known confounders. Significant variables at p-value ≤ 0.10 and modified appropriate categories are considered for multivariate logistic regression analysis after checking co-linearity among the explanatory variables. Forward stepwise logistic regression methods are applied to assess significant explanatory variables of under-five mortality for each birth order. Cross-validation of confidence intervals calculated for odds ratios derived by multivariate logistic regression analysis are internally validated via bootstrapping re-sampling methods using 100re-samples for each birth order model (Kleinbaum et al. 1998). The developed model’s ability to discriminate was assessed using Area Under the Receiver Operating Characteristic (AUROC) (Bewick et al. 2005). The AUROC, ranging from 0.5 to 1.0, justifies the probabilistic model by describing its ability to reliably predict the event, in this case, under-five mortality. P-value ≤ 0.05 (two tailed) is considered for statistical significant level. SPSS 20.0 statistical package is used for the analysis (SPSS^2011^).

## Results

Child status (live or dead) is taken as dependent variable and percentage of deaths in parenthesis with distribution of each category of variable for each birth order are provided in Table 1. Mother’s average age for birth order 1 to 5 are 23.7 years, 25.8 years, 27.4 years, 29 years and 31 years, respectively. Most mothers whose children died are Hindu, with no formal education, severely anaemic and working in the agricultural sector. A regional distribution of under-five mortality shows that it is higher for birth order 1 than 2 in most regions. However, under-five mortality is higher in Central and East regions than in North, North East, West and South regions for all birth orders.

**Table 1:** **Distribution of covariates across birth order (1–5) of children and their percentage of deaths in parenthesis**

Variables	Category	Birth order				
	Total	1 (16567)	2 (14409)	3 (8318)	4 (5021)	5 (3034)
Mother’s age at index child	Continuous	23.7 ± 4.2	25.8 ± 4.3	27.4 ± 4.3	29 ± 4.3	31 ± 4.5
Gender of child	Male	8474(6.3)	7521(4.4)	4447(5)	2621(5.6)	1556(5.8)
	Female	8093(5)	6888(4.6)	3871(5.1)	2400(7.0)	1478(8.3)
Place of residence	Rural	9282(6.9)	8366(5.2)	5429(5.4)	3484(6.5)	2246(6.9)
	Urban	7285(4.1)	6043(3.5)	2889(4.3)	1537(5.8)	788(7.2)
Religion	Hindu	12062(5.8)	10409(4.7)	5678(5.1)	3213(6.9)	1876(7.7)
	Muslim	2231(6)	2062(3.9)	1450(5.8)	1026(4.1)	660(5.3)
	Christian	1431(3.9)	1238(3.9)	827(3.6)	585(6.2)	377(6.1)
	Others	830(5.2)	684(3.5)	358(4.5)	191(6.8)	115(8.7)
Type of caste	Scheduled caste	2759(6.8)	2543(5.9)	1591(5.9)	962(7.3)	594(7.9)
	Scheduled tribe	2204(6.7)	1924(5.2)	1437(5.0)	1074(7.1)	705(6.5)
	Other backward classes	5298(6.0)	4714(4.4)	2794(4.8)	1644(6.0)	1038(7.2)
	Others	5634(4.4)	4676(3.5)	2140(4.5)	1122(4.9)	586(6.8)
Wealth index	Poorest	1890(10.8)	1872(7.2)	1597(6.2)	1303(8.1)	969(7.8)
	Poorer	2412(8.7)	2287(7.0)	1713(7.2)	1194(5.9)	776(5.4)
	Middle	3231(6.5)	2945(4.4)	1854(4.4)	1115(4.9)	649(8.3)
	Richer	4048(4.1)	3569(3.8)	1859(3.8)	930(6.9)	444(4.1)
	Richest	4986(2.9)	3736(2.2)	1295(3.5)	479(4.0)	196(7.7)
Standard of living	Low	2554(9.8)	2677(6.9)	2184(6.7)	1745(7.4)	1232(8.4)
	Medium	4531(6.4)	4302(5.1)	2888(4.6)	1814(6.1)	1122(6.6)
	High	7372(3.7)	6077(2.5)	2642(3.9)	1152(5.4)	566(5.8)
Mother’s education	No formal education	4122(9.4)	4468(7.4)	3879(6.1)	3099(6.8)	2143(7.4)
	Primary	2242(7.4)	2160(5.1)	1438(5.6)	742(5.7)	405(6.2)
	Secondary	8058(4.2)	6374(2.9)	2721(3.5)	1112(5.1)	471(6.2)
	Higher	2144(2.1)	1407(1.2)	280(2.5)	68(4.4)	15(6.7)
Father’s education	No formal education	2409(9.7)	2598(6.9)	2180(5.7)	1734(6.8)	1286(7.4)
	Primary	2016(7.9)	1967(6.2)	1362(5.9)	892(6.7)	531(7.7)
	Secondary	8944(4.9)	7532(4.0)	4037(4.7)	2091(5.9)	1073(6.5)
	Higher	3010(3.0)	2171(1.9)	645(3.1)	247(3.6)	100(4.0)
Mother’s occupation	Non-working	12080(5.1)	9836(3.8)	5059(4.8)	2770(5.8)	1531(6.5)
	Service	2079(5.2)	1995(5.1)	1192(5.6)	770(7.0)	472(8.9)
	Agriculture	2400(8.8)	2564(6.5)	2057(5.4)	1480(6.8)	1031(6.9)
Father’s occupation	Non-working	243(6.6)	133(2.3)	75(5.3)	45(4.4)	18(11.1)
	Service	12626(5.1)	10908(4.2)	5881(5.1)	3336(6.2)	1904(7.1)
	Agriculture	3610(7.5)	3315(5.5)	2332(4.7)	1625(6.4)	1100(6.8)
Mother’s anemia level	Severe	218(7.8)	211(10.9)	134(11.2)	98(10.2)	70(12.9)
	Moderate	2415(7.7)	2119(6.6)	1222(6.5)	770(8.3)	518(9.7)
	Mild	5801(5.9)	5050(4.4)	3045(5.2)	1873(5.8)	1094(5.9)
	Not anaemic	6603(5.0)	5729(3.8)	3085(4.1)	1727(5.6)	1000(6.7)
Prenatal care	No	945(5.8)	1194(5.1)	1271(3.5)	1040(4.6)	828(3.9)
	Yes	944(3)	4676(3.5)	2140(4.5)	1122(4.9)	586(6.8)
Antenatal care	Home	757(4.5)	880(2.3)	538(3.5)	308(2.6)	178(5.1)
	Govt. hospital	3876(3.4)	4432(2.8)	2598(3)	1499(3.6)	780(5.4)
	Private hospital	4606(2.4)	4219(1.9)	1752(2.2)	858(4.2)	433(3.5)
	Village/NGO	188(3.7)	188(2.7)	126(0.8)	53(1.9)	33(3.0)
Information regarding complications in pregnancy	No	6294(3.6)	6735(2.4)	3746(2.8)	2103(3.9)	1193(4.9)
	Yes	3148(1.9)	2993(2.2)	1276(2.4)	619(3.1)	233(3.9)
Pregnancy health Nutrition education	No	1574(4.1)	1371(3.6)	915(3.6)	534(5.1)	352(5.4)
	Yes	1474(4.6)	1368(3.4)	765(3.3)	415(3.6)	211(4.7)
Breastfeeding health nutrition education	No	1300(1.9)	1131(2.2)	764(2.1)	450(2.4)	279(4.3)
	Yes	1090(1.5)	1049(2.4)	582(2.2)	309(2.3)	163(5.5)
Size of child at birth	Very large	592(6.6)	577(3.8)	308(4.9)	191(6.8)	109(2.8)
	Larger than average	3181(4.8)	2848(3.9)	1519(4.1)	880(3.9)	527(6.3)
	Average	9040(4.5)	8008(3.6)	4725(4.4)	2853(5.3)	1689(6.7)
	Smaller than average	2425(6.2)	1932(5.4)	1140(6.1)	705(5.3)	442(7.5)
	Very small	1056(10.9)	801(11.4)	467(10.7)	295(15.3)	211(13.3)
Place of delivery	At home	6115(7.9)	7019(5.7)	5406(5.5)	3716(6.1)	2423(6.8)
	At Hospital/others	10418(4.1)	7372(3.2)	2906(4.2)	1300(6.2)	607(7.6)
Assistance at delivery	No	35(25.7)	58(3.4)	64(3.1)	53(9.4)	53(0)
	Yes	16495(5.4)	14327(4.4)	8247(5)	4961(6.1)	2976(7.1)
Breastfeeding	No	5457(9)	5474(6.5)	2942(8.0)	1762(10.2)	1034(12.3)
	Yes	11110(4.0)	8935(3.2)	5376(3.4)	3259(4.1)	2000(4.3)
Immunization	No	7333(5.3)	6099(4.4)	3260(5.1)	1785(6.7)	1029(6.7)
	Yes	1813(4.1)	1296(2.5)	595(4.0)	287(6.3)	153(5.2)
Child age in months	<1 month	3844(16.9)	3127(12.8)	1725(12.7)	1055(15.6)	627(17.4)
	1- 12 months	3390(6.7)	2993(6.3)	1720(8.5)	912(11.6)	609(11.7)
	> 12 months	9333(0.6)	8289(0.7)	4873(1.1)	3054(1.4)	1798(1.8)
Birth interval	< 24 months	---	4204(6.5)	2197(7.9)	1291(10.1)	756(11.8)
	24-36 months	---	4707(4.0)	2964(4.5)	1833(5.7)	1150(6.9)
	>36 months	---	5380(3.1)	3152(3.6)	1896(4.1)	1128(4.0)
Region	North	3009(4.8)	2717(4.3)	1589(5)	856(6.5)	487(6)
	Central	2969(8.9)	2744(6)	1978(7.4)	1428(7.4)	989(9.8)
	East	2566(7.3)	2083(5.2)	1301(5.4)	854(5.6)	541(5.9)
	North East	2965(4.5)	2422(4.6)	1573(4.7)	1083(6.5)	628(5.6)
	West	2191(3.8)	1825(3.6)	832(4.2)	389(5.7)	214(4.7)
	South	2867(4.3)	2618(3)	1045(3.8)	411(3.2)	175(5.7)

Unadjusted odds ratios and 95% CI of Univariate analysis are presented in Table 2. In Birth order 1, living in rural area increases the risk to under-five mortality by 73% (OR, 1.73; 95% CI, 1.50 - 2.00) compared to urban living areas. Increased wealth index and standard of living increases the chance of under-five survival significantly. Compared with no formal education, the higher the level of mother’s education and father’s education, the greater is the chance of under-five survival. Mother’s occupation in agriculture increases the risk of under-five mortality by 80% (OR, 1.79; 95% CI, 1.52 - 2.10). Female child has 22% (OR, 0.78; 95% CI, 0.68 - 0.89), Christians have 34% (OR, 0.66; 95% CI, 0.50 - 0.87) and all other castes have a better chance of surviving than males, Hindus and scheduled castes, respectively. Breastfeeding (OR, 0.42; 95% CI, 0.37 – 0.48) and immunization (OR, 0.76; 95% CI, 0.60 – 0.98) are protective factors. Breastfeeding health nutrition education increases survival by 24% (OR, 0.76; 95% CI 0.40 – 1.43). Children of non-anaemic mothers have a 38% (OR, 0.62; 95% CI, 0.37 – 1.02) better chance of survival than mothers with severe anaemia. Hospital delivery, prenatal care and information regarding complications in pregnancy increase the chance of under-five survival compared to home delivery, no care, and no information regarding complications in pregnancy respectively. A below average size of child at birth significantly increases the chance of mortality than an average or above average size of child at birth. Antenatal care increases the chance of survival than no care. Private hospitals perform better than governmental facilities.

**Table 2:** **Univariate analysis of child mortality across birth order (1–5)**

Variables	Categories	Birth order−1	Birth order −2	Birth order −3	Birth order −4	Birth order −5
		Unadjusted	Unadjusted	Unadjusted	Unadjusted	Unadjusted
		OR & CI 95%	OR & CI 95%	OR & CI 95%	OR & CI 95%	OR & CI 95%
Mother’s age	Continuous	0.93(0.91– 0.94)	0.93(0.91- 0.95)	0.95(0.93- 0.98)	0.97(0.95 - 0.99)	0.99(0.96 - 1.02)
Gender of child	Female	0.78(0.68 – 0.89)	1.04(0.89– 1.21)	1.03(0.85– 1.26)	1.26(1.01– 1.58)	1.48(1.12– 1.96)
Place of residence	Rural	1.73(1.50 – 2.00)	1.50(1.27– 1.78)	1.27(1.02– 1.57)	1.12(0.87– 1.45)	0.96(0.69– 1.31)
Religion	Muslim	1.03(0.85 – 1.25)	0.81(0.64– 1.03)	1.15(0.89– 1.47)	0.57(0.41– 0.80)	0.67(0.46– 0.98)
	Christian	0.66(0.50 – 0.87)	0.81(0.60– 1.10)	0.81(0.48– 1.03)	0.88(0.61– 1.27)	0.78(0.49– 1.22)
	Others	0.88(0.65 – 1.22)	0.73(0.48– 1.11)	0.73(0.52– 1.46)	0.98(0.55– 1.75)	1.14(0.58– 2.22)
Caste	Scheduled tribe	0.97(0.78 – 1.22)	0.87(0.67– 1.12)	0.84(0.61– 1.15)	0.97(0.70– 1.36)	0.81(0.53– 1.24)
	Other backward classes	0.87(0.72 – 1.05)	0.74(0.59– 0.91)	0.80(0.61– 1.04)	0.82(0.60– 1.12)	0.91(0.62– 1.33)
	Others	0.62(0.51 – 0.76)	0.58(0.46– 0.73)	0.75(0.56– 1.10)	0.66(0.46– 0.95)	0.85(0.55– 1.32)
Wealth index	Middle	0.65(0.55 – 0.77)	0.61(0.49 – 0.75)	0.64(0.49 – 0.83)	0.68(0.50 – 0.93)	1.17(0.84 – 1.63)
	Richer & richest	0.33(0.28 – 0.39)	0.40(0.34 – 0.48)	0.53(0.42 – 0.67)	0.63(0.63 – 1.08)	0.70(0.47 – 1.04)
Standard of living	Medium	0.63(0.52 – 0.75)	0.72(0.60 – 0.88)	0.67(0.53 – 0.86)	0.80(0.62 – 1.04)	0.77(0.57 – 1.06)
	High	0.36(0.30 – 0.43)	0.35(0.28 – 0.43)	0.57(0.44 – 0.74)	0.71(0.52 – 0.97)	0.68(0.45 – 1.02)
Mother’s education	Primary	0.76(0.63 – 0.92)	0.67(0.54 – 0.84)	0.90(0.70 – 1.17)	0.82(0.58–1.15)	0.83(0.53– 1.28)
	Secondary	0.42(0.36 – 0.49)	0.38(0.31 – 0.45)	0.55(0.40 – 0.69)	0.74(0.55– 0.99)	0.82(0.55– 1.24)
	Higher	0.21(0.15 – 0.28)	0.15(0.09 – 0.25)	0.39(0.10 – 0.84)	0.63(0.20– 2.01)	0.89(0.12– 6.87)
Father’s education	Primary	0.80(0.65 – 0.98)	0.89(0.70 – 1.13)	1.04(0.78 – 1.39)	0.99(0.72 – 1.36)	1.05(0.71 – 1.54)
	Secondary	0.48(0.41 – 0.57)	0.56(0.46 – 0.68)	0.82(0.65 – 1.03)	0.86(0.66 – 1.11)	0.88(0.64 – 1.21)
	Higher	0.29(0.22 – 0.37)	0.27(0.20 – 0.38)	0.53(0.33 – 0.85)	0.52(0.26 – 0.03)	0.52(0.18 – 1.45)
Mother’s occupation	Service	1.02(0.83 – 1.26)	1.35(1.08 – 1.69)	1.19(0.90 – 1.57)	1.23(0.90– 1.70)	1.40(0.96– 2.04)
	Agriculture	1.79(1.52 – 2.10)	1.75(1.45 – 2.11)	1.14(0.91 – 1.44)	1.18(0.91– 1.53)	1.06(0.77– 1.45)
Father’s occupation	Service	0.76(0.46 – 1.28)	1.90(0.60 – 6.00)	0.94(0.34 – 2.65)	1.43(0.34– 5.94)	0.65(0.14 – 2.70)
	Agriculture	1.15(0.68 – 1.93)	2.53(0.80 – 8.00)	0.88(0.32 – 2.45)	1.47(0.35– 6.15)	0.59(0.13 – 2.60)
Mother’s anaemia	Moderate	0.99(0.50 – 1.66)	0.57(0.36 – 0.91)	0.56(0.31 – 0.99)	0.80(0.40– 1.61)	0.72(0.34– 1.55)
	Mild- anaemia	0.74(0.45 – 1.23)	0.38(0.24 – 0.59)	0.43(0.25 – 0.76)	0.54(0.27– 1.07)	0.42(0.20– 0.89)
	No- anaemia	0.62(0.37 – 1.02)	0.32(0.21 – 0.51)	0.34(0.19 – 0.60)	0.52(0.26– 1.04)	0.49(0.23– 1.02)
Prenatal care	Yes	0.50(0.37 – 0.68)	0.45(0.34 – 0.60)	0.78(0.55 – 1.10)	0.79(0.55 – 1.12)	1.23(0.80 – 1.88)
Antenatal care	Govt. hospital	0.75(0.51 – 1.10)	1.20(0.78 – 2.03)	0.83(0.50 – 1.36)	1.40(0.66 – 2.98)	1.07 (0.51– 2.24)
	Private hospital	0.53(0.36 – 0.78)	0.83(0.51 – 1.36)	0.62(0.36 – 1.09)	1.64(0.76 – 3.57)	0.67(0.29 – 1.57)
	Village/NGO	0.82(0.36 – 1.88)	1.18(0.44 – 3.17)	0.21(0.03 – 1.65)	0.72(0.09 – 5.89)	0.59(0.07 – 4.79)
Information regarding complications in pregnancy	Yes	0.52(0.39 – 0.70)	0.90(0.67 – 1.20)	0.83(0.53 – 1.25)	0.80(0.48– 1.31)	0.79(0.38 – 1.61)
Pregnancy health nutrition education	Yes	1.12(0.80 – 1.60)	0.94(0.63 – 1.41)	0.90(0.53 – 1.53)	0.70(0.37 – 1.34)	0.87(0.40 – 1.91)
Breastfeeding health nutrition education	Yes	0.76(0.40 – 1.43)	1.08(0.62 – 1.89)	1.07(0.51 – 2.34)	0.93(0.36 – 2.41)	1.30(0.54 – 3.16)
Size of child at birth	Average	0.88(0.74 – 1.05)	0.92(0.75 – 1.14)	1.02(0.78 – 1.33)	1.23(0.88 – 1.71)	1.40(0.94 – 2.12)
	Below average	1.54(1.27 – 1.86)	1.93(1.54 – 2.42)	1.80(1.34 – 2.40)	2.56(1.80 – 3.65)	2.00(1.29 – 3.14)
Place of delivery	Hospital	0.50(0.44 – 0.57)	0.54(0.46 – 0.64)	0.75(0.61 – 0.94)	1.02(0.78 – 1.32)	1.13(0.80 – 1.59)
Breastfeeding	Yes	0.42(0.37 – 0.48)	0.48(0.41 – 0.56)	0.40(0.33 – 0.49)	0.38(0.30 – 0.48)	0.32(0.24 – 0.43)
Immunization	Yes	0.76(0.60 – 0.98)	0.57(0.40 – 0.82)	0.78(0.51 – 1.21)	0.92(0.56 – 1.55)	0.77(0.36 – 1.63)
Child age in months	1-12 months	0.35(0.30 – 0.41)	0.60(0.50 – 0.73)	0.64(0.52 – 0.80)	0.70(0.55 – 0.92)	0.63(0.45 – 0.87)
	>12 month	0.03(0.20 – 0.04)	0.46(0.38 – 0.56)	0.08(0.05 – 0.10)	0.07(0.05 – 0.11)	0.09(0.06 – 0.13)
Birth interval	24-36 months	--	0.60(0.50 – 0.73)	0.54(0.41 – 0.70)	0.53(0.41 – 0.70)	0.55(0.40 – 0.76)
	>36 months	--	0.46(0.38 – 0.56)	0.43(0.34 – 0.55)	0.38(0.28 – 0.51)	0.31(0.22 – 0.45)

Reference Category: Gender of Child: Male; Place of residence: Rural; Religion: Hindu; Caste: Schedule Caste; Wealth Index: Poorer & poorest; Standard of living: Low; Women education: No formal education; Father Education: No formal Education; Mother Occupation: Non-Working; Father Occupation: Non-Working; Mother Smoking: No; Mother Anemia: Severe; Prenatal Care: No; Antenatal Care: Home; Information regarding complications in pregnancy: No; Pregnancy Health Nutrition Education: No; and Breastfeeding Health Nutrition: No. Size of Child at birth: Above average; Place of delivery: Home; Breastfeeding: No; Immunization: No; Pregnancy Complication: No and Birth Interval: <24 months. ; Child age in months: < 1 month and Birth Interval: < 24 months.

In Birth Order 2, mother’s education and occupation, mother’s anaemia, place of residence, caste, breastfeeding, immunization, wealth index, father’s education, standard of living, place of delivery, size of child at birth, prenatal care and previous birth interval greater than 2 years are significant factors. In Birth Order 3, mother’s education, religion, mother’s anaemia, place of residence, caste, breastfeeding, wealth index, father’s education, standard of living, place of delivery, size of child at birth and birth interval are significant factors.

In Birth order 4, mother’s education, religion, gender, breastfeeding, wealth index, standard of living, size of child at birth and birth interval are statistically significant. Breastfeeding, wealth index and standard of living increase the chance of under-five survival. A below average size of child at birth increases the risk of dying by two and a half times compared to an above average size of child at birth (OR, 2.56, 95% CI, 1.80-3.65). A birth interval of greater than 24 months increases under-five survival significantly. Muslim religion acts as a protective factor in under-five survival compared to Hindu religion. Female children are at a 26% (OR, 1.26; 95% CI, 1.01 – 1.58) greater risk of dying than male children.

In Birth Order 5, religion, mother’s anaemia, gender, breastfeeding, size of child at birth and birth interval are statistically significant. The most notable absences at birth order 5 are mother’s and father’s education. Muslims have a 33% (OR, 0.67; 95% CI, 0.46– 0.98) better chance of survival than Hindus. Others have a risk factor at 14% (OR, 1.14; 95% CI, 0.58– 2.22) compared to Hindus but is not statistically significant. Mother’s anaemia effects under-five survival inversely. A female child is at 48% (OR, 1.48; 95% CI, 1.12– 1.96) greater risk of mortality than a male child. Breastfeeding increases the chance of under-five survival. A below average size of child at birth increases the risk by 2 times (OR, 2.00; 95% CI, 1.29 – 3.14) compared with above average size of child at birth. A birth interval greater than 24 months increases under-five survival compared to lesser birth interval.

Most fathers whose children died are poor, with low standard of living, no formal education and working in agricultural sector for birth order 1 and 2. However, fathers either working or in agriculture had similar effects for birth order 3 to 5. There are very few fathers who are non-working and this did not show any effect on under-five mortality.

Adjusted odds ratios with 95% CI for multivariate analysis are provided in Table 3 for each birth order model. Multivariate model for birth order 1 has female, higher mother’s education, Christian and richer and richest wealth index as significant protective factors. Breastfeeding (OR, 0.04, 95% CI, 0.03-0.05) and prenatal care (OR, 0.04, 95% CI, 0.03-0.06) improve under-five survival by 96%. Secondary mother’s education, Muslim, middle wealth index and information regarding complications in pregnancy are also protective factors. Mother’s occupation in service (OR, 1.25, 95% CI, 0.92-1.70) or agriculture (OR, 1.35, 95% CI, 1.05-1.73) is a risk factor. Multivariate model for birth order 2 has place of delivery, primary and above mother’s education, richer and richest wealth index, immunization and previous birth interval greater than 24 months as highly significant factors in under-five survival. Mother’s occupation in service (OR, 1.89, 95% CI, 1.43-2.50) or agriculture (OR, 1.51, 95% CI, 1.20-1.93) are risk factors. Multivariate model for birth order 3 has secondary mother’s education, middle and richer and richest wealth index, breastfeeding and previous birth interval greater than 24 months as highly significant factors. Multivariate model for birth order 4 has secondary mother’s education, Muslim religion, breastfeeding and previous birth interval greater than 24 months as highly significant factors. Below average child size at birth is a significant risk factor (OR, 2.20, 95% CI, 1.42-3.34). Multivariate model for birth order 5 has richer and richest wealth index, breastfeeding and previous birth interval greater than 24 months as protective significant factors for under-five survival. Female gender of child is a significant risk factor (OR, 1.61, 95% CI, 1.16-2.23).

**Table 3:** **Multivariate analysis of child mortality across birth order (1–5)**

Variables	Categories	Birth order −1	Birth order −2	Birth order −3	Birth order −4	Birth order −5
		Adjusted	Adjusted	Adjusted	Adjusted	Adjusted
		OR & CI 95%	OR & CI 95%	OR & CI 95%	OR & CI 95%	OR & CI 95%
Gender of child	Female	0.76(0.63 – 0.91)				1.61(1.16– 2.23)
Religion	Muslim	0.84(0.63 – 1.12)			0.53(0.35 – 0.88)	
	Christian	0.44(0.30 – 0.64)			0.70(0.44 – 1.10)	
	Others	0.70(0.45 – 1.08)			1.00(0.49 – 2.00)	
Wealth index	Middle	0.87(0.67 – 1.12)	0.80(0.61 – 1.03)	0.56(0.41 – 0.77)	0.61(0.42 – 0.90)	0.91(0.61 – 1.34)
	Richer & richest	0.61(0.47 – 0.79)	0.59(0.45 – 0.77)	0.43(0.31 – 0.60)	0.42(0.86 – 1.24)	0.45(0.28 – 0.71)
Mother’s education	Primary	1.07(0.82 – 1.41)	0.66(0.50 – 0.87)	0.90(0.66 – 1.24)	0.57(0.37 – 0.88)	
	Secondary	0.79(0.62 – 1.01)	0.37(0.29 – 0.48)	0.53(0.38 – 0.73)	0.53(0.35 – 0.79)	
	Higher	0.31(0.20 – 0.50)	0.09(0.05 – 0.16)	0.35(0.15 – 0.83)	0.20(0.04 – 0.95)	
Mother’s occupation	Service	1.25(0.92 – 1.70)	1.89(1.43 – 2.50)			
	Agriculture	1.35(1.05 – 1.73)	1.51(1.20 – 1.93)			
Prenatal care	Yes	0.04(0.03 – 0.06)				
Information regarding complications in pregnancy	Yes	0.66(0.47 – 0.92)				
Size of child at birth	Average				1.37(0.92 – 2.03)	
Place of delivery	Hospital		0.72(0.58 – 0.91)			
	Below average				2.20(1.42 – 3.34)	
Breastfeeding	Yes	0.04(0.03– 0.05)	0.08(0.06 – 0.10)	0.08(0.06–0.11)	0.07(0.44 – 0.90)	0.08(0.05 – 0.11)
Immunization	Yes		0.44(0.30 – 0.65)			
Birth interval	24-36 months	--	0.56(0.45 – 0.70)	0.45(0.34 – 0.60)	0.50(0.37 – 0.70)	0.50(0.34 – 0.73)
	>36 months	--	0.35(0.28 – 0.45)	0.30(0.22 – 0.42)	0.25(0.17 – 0.36)	0.21(0.14 – 0.35)

Reference Category: Gender of Index Child: Male; Religion: Hindu; Wealth Index: Poorer & poorest; Mother’s education: No formal education; Mother Occupation: Non-Working; Prenatal Care: No; Information regarding complications in pregnancy: No; Place of delivery: Home; Breastfeeding: No; Immunization: No and Birth Interval: <24 months.

Note: Mother’s age and child’s age in months are forced into multivariate analysis at each birth order.

**Figure 1:**
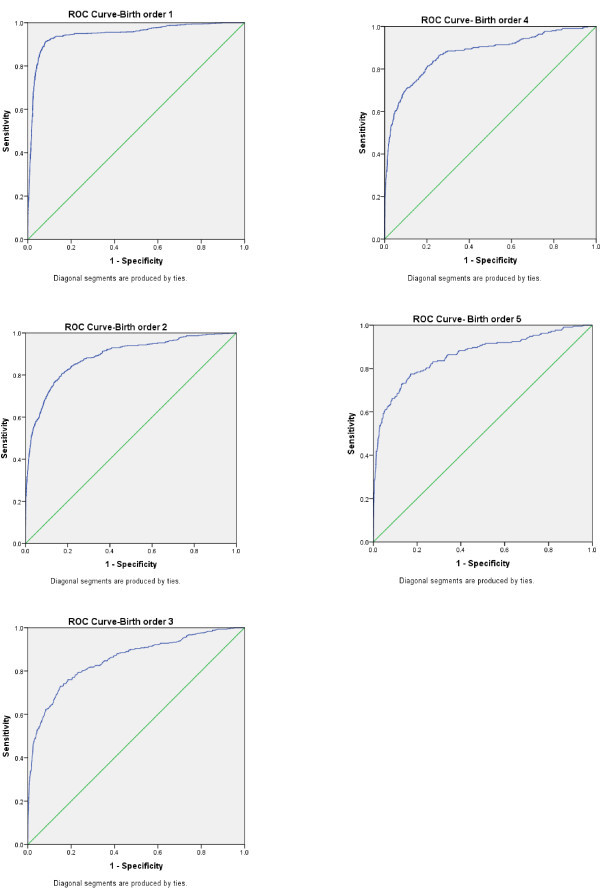
**ROC Curves for birth order 1 to 5.**

Bootstrapping analysis showed negligible bias in estimated parameters. The ROC curves for different models are shown in Figure 1. The AUROC was 0.94 (95% CI, 0.93-0.96), 0.89(0.87 – 0.90), 0.86 (0.84 – 0.88), 0.85 (0.82 – 0.88) and 0.84 (0.81 – 0.87) for the developed models 1 to 5, respectively.

## Discussion

Increasing maternal age is a protective factor in under-five survival. Average maternal age is significantly high for under-five survival in birth order 1 to 5. This is a good sign in a country that has been fraught with issues of child marriages and early and/ or unwanted pregnancies. Almost half of 20–24 year old women in India (44.5%) are married before age 18, and 22% of all 20–24 year old women have given birth by age 18 years (NFHS-3 2007). However, our study shows a mean age at birth order 1 to be 23.7 years at the national level which means that awareness has improved later pregnancies in many other populations.

The effects of breastfeeding and birth interval greater than 24 months are significant protective factors from birth order 2 to 5. This is in line with the general perception and is a well- documented fact (Robert et al. 2003; WHO 2000; Gareth et al. 2003; Singh et al. 2012a; Singh et al. 2012b). The UNICEF report projects that if all birth to pregnancy intervals were 3 years, approximately 1.6 million under-five deaths could be prevented annually (UNICEF 2012). Efforts should continue to delay the next pregnancy.

Mother’s education is a protective factor for birth order 1 to 4. However, it did not emerge as a significant factor in birth order 5 because socio-cultural factors other than education dominate the couple’s choice of having children. This may be explained by the fact that most families decide their fertility choices early on and hence education plays a relatively small role at higher birth order for under-five survival. However, maternal education plays an important part in utilization of antenatal care (Kumar et al. 2013; Chalasani 2012; Mazumdar 2010; Pradhan et al. 2010; Gakidou et al. 2010; Boone et al. 2006).

Mothers’ working in agriculture sector is a risk factor in birth order 1 and 2 in univariate and multivariate analysis. Working mothers are also at risk in birth order 2. However, there was no significant risk in birth order 3 to 5. Women should be advised against heavy labour especially in the rural agricultural sector. Headey et al. (2012) noted that agricultural workers have the lowest BMIs compared with non-agricultural workers, even after controlling for wealth, health, education, and location. Another study from Kenya by Mustafa and Odimegwu (2008) reports a higher rate of under-five mortality for mothers working in agriculture compared to those not working. Our study specifically identifies mothers at birth order 1 and 2 working in agriculture as being at risk. Further studies are needed to see the reasons for the poor survival of children born to mothers working in the agricultural sector.

The univariate analysis reveals that under-five survival is significantly better in Others (that is, higher castes) compared with scheduled castes. However, it is not a statistically significant factor in multivariate analysis for any birth order. Religion acted differentially in different birth orders. Christians have a better chance at survival in birth order 1 and 3 while Muslims survive better at birth order 4 and 5, compared with Hindus. Multivariate analysis supported univariate analysis with Christians and Muslims having better survival than Hindus in birth order 1 and 4, respectively. This agrees with other studies who found that Hindus were disadvantaged in securing the survival of their babies at early ages compared to Muslims and women belonging to other religious groups (Kumar et al. 2013; Bhalotra et al. 2009).

Mothers having severe anaemia are a risk factor to under-five survival across all birth orders in univariate analysis. This reflects the need of early interventions to improve anaemia levels and nutrition index to achieve better results. However, it is not a significant factor in multivariate analysis. Below average size of child is a significant factor in multivariate analysis for birth order 4. The finding is supported by Kumar and colleagues but they did not analyse the factor by birth order (Kumar et al. 2013).

Female children have a better chance of survival than male children in birth order 1. Gender has no visible distinction in birth order 2 and 3, but female children are considerably at risk of dying in birth order 4 and 5. The sex of the previous child or children is unfavourable for the female child (Sawyer 2012; Arokiasamy 2004). This sustains the idea that couples often desire at least one male child and the risk of female child dying at birth order 4 and 5 is very high. In multivariate analysis, female child has a better chance of survival in birth order 1 while it is a risk factor in birth order 5. This is a matter of grave concern since other researchers (Jha et al. 2006) have also noted the increasing discrepancies in sex ratios in both urban and rural areas. Another study reported that all-cause mortality rate in children aged 1–59 months was about 36% higher in girls than in boys and most of the leading causes of death were between 12% and 72% higher in girls than in boys, with the exception of injuries and meningitis/ encephalitis (Bassani et al. 2010). These facts show the need to identify and counsel parents at higher birth order against gender bias.

Wealth inequalities have been shown to effect under-five survival (Chalasani 2012; Mazumdar 2010; Pradhan et al. 2010; Houweling 2012). Under-five mortality is significantly higher in middle, richer and richest wealth index compared to those in poor and poorest wealth index for birth order 1 to 3. However, it is not statistically significant in birth order 4 and 5. It is the same with standard of living. However, wealth index is a protective factor in multivariate analysis for all birth orders. This is important since wealth or economic well-being of the family effects general health of mother and child. It is important to counsel and follow-up those in the lesser privileged sections of society in order to reduce under-five mortality.

Place of birth in rural areas produces risk for birth order 1 to 3 but is not significantly higher for birth order 4 and 5. It strengthens the notion that under-five mortality is as high in rural as urban areas for higher birth orders. It shows the need for improving counselling in urban centres and facilities in rural centres. Neonatals are more at risk of dying than children who survive greater than 1 month and greater than 1 year. Other studies also link higher mortality risk among neonatals with early childbearing trends and lower utilization of maternity services (Kumar et al. 2013; Hall 2005). Our study supports that neonatals are at the highest risk in all birth orders.

Breastfeeding health nutrition education has no effect in birth order 2, 3, 4 and 5. Pregnancy health nutrition education has no effect in birth order 1 but improved under-five survival for all subsequent birth orders. However, neither factor is significant in multivariate analysis though this may be due to large number of missing observations. However, efforts to increase these educational indicators should continue to improve under-five survival.

Under-five survival is better in hospitals than home deliveries for birth order 1 to 3 but is not statistically significant in birth order 4 and 5. However, there is proportional rise in mortality in hospitals and other facilities for birth order 4 and 5, maybe due to complications or due to delay in getting to the hospital. Under-five survival is significantly better in birth order 1 for antenatal care in private hospitals compared to home deliveries. However, the factor was not statistically significant in any other birth order. It has been noted that three-quarters of births in rural India continue to take place at home. Mishra and Retherford have also noted that institutional antenatal care and assistance at delivery have a large effect on under-five mortality (Mishra et al. 2008). Information regarding complications in pregnancy is a protective factor in birth order 1 with under-five survival being better in those who suffer a complication. This is also borne out in multivariate analysis since information regarding complications in pregnancy leads to antenatal interventions and better care. Prenatal care is a significant factor in under-five survival for birth order 1 and 2. It shows improvement in birth order 3 and 4 but is not statistically significant. It is a significant variable for birth order 1 in multivariate analysis. This is an important finding since it indicates that prenatal care makes a difference in under-five survival. This is in conjunction with established literature that states the dangers are higher for nulliparous compared to higher parities (Gubhaju 1985; Nankabirwa et al. 2011).

Apart from these maternal factors, our study also indicates the importance of father’s education in under-five survival. The higher the father’s education, the better the chances of survival for the child from birth order 1 to 5. It is statistically significant that under-five survival is better in secondary and higher educated fathers for birth order 1 to 3 compared to no formal education and primary educated. This is a pointer to the need to educate fathers who are illiterate and/ or studied up to primary level in matters of reproductive and child health. This is supported by a study by Boone and Khan (Boone et al. 2006). However, it is not a significant factor in multivariate analysis. Father’s occupation is not statistically significant in any birth order since it does not directly affect maternal or child health.

The AUROC is used to see if the model fits the data. Prediction models demonstrated high discrimination with a value of 0.91 indicating models fit the data. This demonstrates that the developed models are able to reliably predict the association between exposure variables and maternal factors contributing to under-five mortality extracted from NFHS-3 survey data.

### Strengths

The study captures 92% of the total births during 59 months preceding the survey for the analysis. Thematically, the study is new in segregating information according to birth order (1 to 5) to review the determinants of under-five mortality with a view to improve maternal health care services in India. The predictive accuracy of developed models was above 80% for all developed models indicating a high accuracy to predict the outcome.

### Limitations

The data is based upon recall by the mother and is open to recall biases and undocumented social pressures that lead them to say the “right” thing. The effects of exclusive breastfeeding and total versus partial immunization could not be commented upon due to the restrictions of including breastfeeding and immunization as yes and no dichotomous variables in the analysis. Nutritional differences could also be responsible for differences in under-five survival in religious groups, Christians and Muslims faring better than Hindus. However, our study did not establish a direct link between nutrition and religion though it noted that pregnancy health nutrition education acted as a protective factor. Although contribution of the explanatory variables are statistically significant, predicting mortality accounted for a Nagelkereke R^2^ of 0.56, 0.37, 0.33, 0.34 and 0.34 for the developed models 1 to 5, respectively, indicating small effect.

### Conclusion and programme implications

The study reveals the determinants of under-five mortality according to birth order. While some factors such as breastfeeding and birth interval greater than 24 months are important in under-five survival, several others such as mother’s education and rural- urban place of residence show weaker relationship at higher birth orders. Efforts are needed to educate young women in matters of reproductive health and the benefits of delaying pregnancies. Maternal education, both in formal and informal sectors, can improve their utilization of antenatal care services. Information regarding complications in pregnancy acts as a protective factor for this reason. Pregnancy Health Nutrition education improves under-five survival for birth orders 2 to 5. It is felt that prenatal care and general anxiousness related to first pregnancy that leads couples to seek early care is important in under-five survival. There is need to educate couples regarding correct nutrition, benefits of breastfeeding and immunization and possible complications so that the couples can decide and take precautions. However, complacency at higher birth orders leads to higher rates of under-five mortality. Higher Wealth index is an important indicator in under-five survival. Most parents are not specific about child’s gender in the first birth order but more girl children die at higher birth orders. This clearly points to how gender preferences play a role in under-five mortality. The study identifies various focus groups for increased counselling such as mothers working in the agricultural sector and severely anaemic mothers. However, the study also shows that under-five mortality is as high in rural as urban areas for higher birth orders. It points to the need of IEC (Information, Education and Communication) network to be further strengthened to reduce under-five mortality across the board for higher birth orders.

### Ethical approval

No identifying information is available in the data precluding the need for any ethical approvals. We wish to thank the DHS for making this data available for our study.

## Endnote

^a^Birth order (or parity) refers to the numerical order of the live birth or foetal death, recorded in relation to all previous issue of the mother, irrespective of whether the issue is a live birth or foetal death or whether pregnancies were nuptial or extra-nuptial.
